# Mitochondrial Quality Control in the Maintenance of Cardiovascular Homeostasis: The Roles and Interregulation of UPS, Mitochondrial Dynamics and Mitophagy

**DOI:** 10.1155/2021/3960773

**Published:** 2021-11-11

**Authors:** Yujie Song, Yuerong Xu, Yingying Liu, Jie Gao, Lele Feng, Yuxi Zhang, Lei Shi, Miao Zhang, Dong Guo, Bingchao Qi, Mingming Zhang

**Affiliations:** ^1^Department of Cardiology, Tangdu Hospital, The Fourth Military Medical University, Xi'an 710000, China; ^2^Department of Cardiovascular Surgery, Xijing Hospital, The Fourth Military Medical University, Xi'an 710000, China; ^3^Department of Orthodontics, School of Stomatology, The Fourth Military Medical University, Xi'an 710000, China; ^4^The College of Life Science, Northwest University, Xi'an 710000, China

## Abstract

Maintenance of normal function of mitochondria is vital to the fate and health of cardiomyocytes. Mitochondrial quality control (MQC) mechanisms are essential in governing mitochondrial integrity and function. The ubiquitin-proteasome system (UPS), mitochondrial dynamics, and mitophagy are three major components of MQC. With the progress of research, our understanding of MQC mechanisms continues to deepen. Gradually, we realize that the three MQC mechanisms are not independent of each other. To the contrary, there are crosstalk among the mechanisms, which can make them interact with each other and cooperate well, forming a triangle interplay. Briefly, the UPS system can regulate the level of mitochondrial dynamic proteins and mitophagy receptors. In the process of Parkin-dependent mitophagy, the UPS is also widely activated, performing critical roles. Mitochondrial dynamics have a profound influence on mitophagy. In this review, we provide new processes of the three major MQC mechanisms in the background of cardiomyocytes and delve into the relationship between them.

## 1. Introduction

Mitochondria are organelles in nucleated eukaryotic cell and provide multiple functions. They serve as a “powerhouse” for the cell and are also involved in several other physiological processes such as programmed cell death, apoptosis, autophagy, metabolism, calcium flux, and innate immunity [1–5]. For heart, mitochondria are particularly essential to embryonic cardiac development, cardiomyocyte differentiation, and contractile function [6, 7]. Damage of mitochondria leads to loss of metabolic homeostasis and further excessive ROS production that evokes more severe cell damage and cell death [8–11].

Due to its high energy demand, the hearts are the most mitochondria-rich organ [11]. In this context, it is vital to maintain the health of mitochondrial population of cardiomyocytes. Multiple studies have found mitochondrial dysfunction as a contribution to the development of many cardiovascular diseases, including diabetic cardiomyopathy, alcoholic cardiomyopathy, ischemia-reperfusion injury, septic cardiomyopathy, cardiovascular aging, atherosclerosis, cardiac hypertrophy, and heart failure [12–20]. Nonetheless, precise regulation of mitochondrial quality control (MQC) seems to be rather vague and complex. In general, mechanisms regulating mitochondrial homeostasis are essential for cells, which are collectively known as MQC. MQC covers a wide range of pathways which forms a closely coordinated network [21]. These mechanisms can be classified into two different levels: molecular level and organelle level.

At the molecular level, mitochondria are evolved with the presence of several quality control mechanisms. In the process of mitochondrial protein import, cytosolic and mitochondrial chaperones such as heat shock protein 60 (HSP60), HSP70, and HSP90 play the major role in preventing newly synthesized polypeptides from misfolding before import, targeting them towards the translocase of the outer mitochondrial membrane (TOM), refolding and sorting them correctly when importing is finished. When the proteins fail to fold correctly or are damaged, timely degradation is needed. The degradation process mainly relies on two mechanisms. Firstly, the ubiquitin-proteasome system (UPS), the main protein quality control mechanism, plays a critical part in selectively degrading damaged proteins of mitochondria. Secondly, mitochondria are also equipped with a set of proteases, which supply an assisting action.

**Figure 1:**
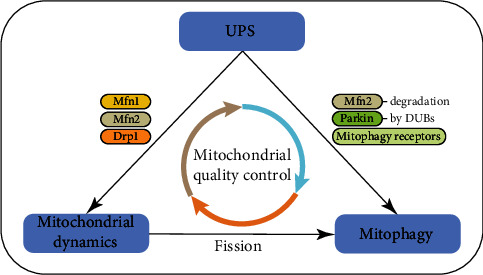
The triangle interplay between the UPS, mitophagy, and mitochondrial dynamics. The UPS influences mitochondrial dynamics by degrading related proteins such as Mfn1, Mfn2, and Drp1. In the same way, UPS regulates receptor-mediated mitophagy. The UPS can also facilitate Parkin-dependent mitophagy by Mfn2 degradation. As components of the UPS, deubiquitinating enzymes (DUBs) are found to play a critical role in regulating the magnitude of Parkin-mediated mitophagy. Finally, mitochondrial fission facilitates mitophagy, but whether fission is a prerequisite for mitophagy in cardiomyocytes remains unclear.

If the damage of mitochondria is beyond the capacity of protein-level mechanisms, the organelle-level mechanisms are activated, including mitochondrial dynamics and mitophagy [22, 23], which will be introduced in detail later. In this review, we will take cardiomyocytes as an example and focus on three major MQC mechanisms: the UPS, mitochondrial dynamics, and mitophagy. These three processes interact with each other and form a triangle interplay (see Figure 1). Thus, we will also delve into describing how these mechanisms can interact with each other.

## 2. UPS

The UPS is the main machinery degrading soluble proteins both in the cytosol and the nucleus. Briefly, damaged proteins are tagged with ubiquitin and delivered to proteasomes for degradation. Ubiquitination of substrates is mediated by three classes of enzymes. Firstly, ubiquitin is activated by the ubiquitin-activating enzyme (E1). Secondly, activated ubiquitin is transferred to a cysteine of an ubiquitin-conjugating enzyme (E2). Thirdly, specific ubiquitin ligases (E3) catalyse the formation of the covalent attachment of ubiquitin to a lysine residue of the target protein. As this reaction repeats, several lysine residues of the target protein are monoubiquitinated, which could be a sufficient signal for the UPS degradation of small proteins [24]. In other cases, ubiquitin chains are built on the substrate as the lysine of ubiquitin itself can also be attached with ubiquitin. Then, the ubiquitin attracted to target protein serves as signals recognized by the 19s regulatory particle of the proteasome, after which the marked protein is degraded [24, 25]. The ubiquitin-dependent modification of substrates can also be reversed by deubiquitinating enzymes (DUBs) [26].

Although proteasome is excluded from mitochondria, an increasing amount of evidence has placed the system as an important contributing factor to the quality control of proteins located not only on the outer mitochondrial membrane (OMM), but also within mitochondria [27, 28].

### 2.1. OMM Proteins

As proteins residing in the OMM are contiguous with the cytosol, they are directly accessible for ubiquitination. There are several E3 enzymes residing in the cytosolic side of the OMM, such as MARCH5 and MULAN/MAPL, which can mediate the ubiquitination process of various OMM substrates, such as mitochondrial adaptor fission 1 (Fis1), dynamin-related protein 1 (Drp1), Mitofusin 1/2 (Mfn1/2), Mcl1, and mitochondrial dynamics proteins of 49 kDa (MiD49). E3 enzymes from other compartments of cell can also be recruited to mitochondrial under certain conditions, among which the best known is Parkin [29].

However, in contrast to soluble proteins, OMM proteins cannot be degraded by proteasome directly after being attached with ubiquitin by E3 enzymes. They have to undergo an extra step termed extraction as they are embedded into the OMM. Similar processes are firstly described in endoplasmic reticulum (ER), which is termed endoplasmic reticulum-associated protein degradation (ERAD) [30]. For ERAD, proteins residing inside ER are translocated to the cytoplasmic side of the ER membrane and then get extracted and be degraded through the proteasome pathway [31, 32]. In mitochondria, a similar system has been proposed, called mitochondria-associated degradation (MAD) [33].

The extraction function of both ERAD and MAD is mainly executed by the ubiquitin-selective chaperone Cdc48 (p97/VCP in mammals) [30, 34]. P97 is a highly conserved ATPase, under the cooperation with its cofactors [35, 36], it can extract polyubiquitinated proteins from various cellular structures, including chromatin, protein assemblies, and membrane. P97 can recognize OMM proteins conjugated with ubiquitin and target them for the degradation by the 26S proteasome [37, 38]. So far, the substrates of MAD that have been reported in the literature are still limited, including Mfn1/2 and Mcl1 [39, 40].

Besides degrading damaged proteins, the UPS also plays a role in adjusting mitochondrial proteome composition, regulating mitochondrial dynamics and maintaining cellular homeostasis [41]. Through regulating mitochondrial dynamics proteins, the UPS can have a profound influence on mitochondrial morphology, which will be described in detail below.

### 2.2. IMM Proteins

Although the UPS is excluded from mitochondria, accumulating evidences have proved that proteins inside the organelle are also substrates of the proteasome mediating degradation [33]. Recently, two proteins reside in the mitochondrial matrix (Kgd1p and Pim1p) are validated to be substrate of the MAD [40]. This is supported by (a) MAD inhibition caused upregulation in ubiquitination and steady-state levels of both proteins and (b) Kgd1p coimmunoprecipitated with Cdc48p, and the interaction is increased by chronic mitochondrial oxidative stress [40]. Other intramitochondria proteins proved to be substrates of MAD include succinate dehydrogenase subunit A (SDHA), UCP2, and UCP3 [42–45].

However, the mechanisms underlying the process of internal mitochondrial protein retrotranslocation still remain largely unknown. It is hypothesized that proteins folded into a stable structure are trapped inside the mitochondrial, which is termed as the fold trap [46]. Unfolded proteins can retrotranslocate to the outside of the organelle, which may be relevant to the MAD process [33]. Besides, it is proposed that translocase of the outer membrane (TOM) complexes may act as an exit channel for MAD [47].

## 3. Mitophagy

Mitophagy is a special kind of autophagy that selectively degrades damaged or redundant mitochondria which serves as a critical mechanism of MQC. Mitophagy plays important physiological roles in regulating and renewing mitochondrial population during many processes such as organism development and cellular differentiation, which is called programmed mitophagy [48, 49]. Recent reports have found that mitophagy can also occur in normal conditions, which is termed basal mitophagy [50–52]. It is believed that tissues with greater level of metabolism such as cardiomyocytes have a higher degree of basal mitophagy. Under pathological conditions, mitophagy acts as a critical mechanism for cell to combat various kinds of stress such as nutrient starvation and hypoxia. Emerging data suggest that dysfunction of mitophagy is involved in the progress of various cardiovascular diseases, including diabetic cardiomyopathy, heart failure, and cardiac aging [53–55].

In general, four main steps are needed to complete mitophagy. Firstly, the target mitochondria are isolated from its network. Secondly, it is marked for degradation. Thirdly, autophagy receptors link the marked mitochondrion with LC3 on the autophagosome, which leads to the formation of autophagosome that engulfs the mitochondria. Finally, the autophagosome fuses with a lysosome, and the cargo inside is degraded by lysosomal enzymes [56]. According to whether ubiquitination is needed in the process of recruiting autophagosome, mitophagy can be divided into two types: ubiquitin-dependent mitophagy and ubiquitin-independent mitophagy.

### 3.1. Ubiquitin-Dependent Mitophagy

The PINK1/Parkin pathway is the most well-studied mechanism of mitophagy, belonging to ubiquitin-dependent type of mitophagy. Serine/threonine kinase PTEN-induced putative kinase 1 (PINK1) serves as a sensor to mark damaged mitochondria. Under normal conditions, PINK1 is continuously transported into the mitochondria and rapidly degraded by proteolysis. However, when mitochondria are damaged (marked by the loss of membrane potential), PINK1 translocation is prevented, causing PINK1 accumulation selectively on the OMM of the damaged mitochondria [57]. Accumulated PINK1 undergoes autophosphorylation at Ser228 and Ser402 and gets activated [58]. Then, activated PINK1 phosphorylates ubiquitin present at a basal level on OMM at Ser65, generating phosphor-ubiquitin. At the same time, Parkin is recruited to the OMM and gets activated through phosphorylation by PINK1. In turn, Parkin polyubiquitinates multiple proteins on the OMM, including Mfn1/2, voltage-dependent anion channel (VDAC), and mitochondrial Rho GTPase-1 (MIRO1) [56, 59]. Newly ubiquitinated proteins are phosphorylated by PINK1, which further promotes Parkin activity. In this way, phosphorylation and ubiquitin modification form a feedforward loop which recruits autophagy receptors to the mitochondria.

The mitophagy receptors possess both ubiquitin-binding domains and LC3-interacting region (LIR) motifs, which enable them to bridge ubiquitinated OMM proteins with the forming autophagosome. Three receptors are thought to play major roles in this model, including Optineurin (OPTN), NDP52 (CALCOCO2), and TAX1BP1 [60].

However, recent progress shows that the role of Parkin in this model is not indispensable. On the contrary, PINK1-mediated phosphorylation of ubiquitin is adequate to recruit autophagy receptors NDP52 and OPTN and induce low-amplitude mitophagy [60].

### 3.2. Ubiquitin-Independent Mitophagy

There are also some mitochondrial receptors that do not require the ubiquitination of OMM proteins to bridge the cargo with the forming autophagosome, which is called the ubiquitin-independent mitophagy. These mitophagy receptors include FUN14 domain containing 1 (FUNDC1), mitochondrial proapoptotic BH3-only domain protein (BNIP3), and BNIP3L/NIX.

The mitophagy receptors reside on the OMM and can directly bind LC3 with their LIR motifs. Current knowledge suggests that ubiquitin-independent receptors can be upregulated or activated by different stimuli in different context. For instance, both BNIP3 and NIX participate in the regulation of mitophagy in adult hearts. BNIP3 upregulation responses to hypoxia, while NIX upregulation responses to G alpha (q)-mediated hypertrophic stimuli [61].

FUNDC1-mediated mitophagy was initially reported to be activated by hypoxia/ischemia [62, 63]. Hypoxia stimulation can induce dephosphorylation of FUNDC1 and enhance its interaction with LC3, which can promote mitophagy. Subsequent studies show that FUNDC1 is closely related to several cardiovascular diseases [64–69]. FUNDC1 is related to cardiac ischemia/reperfusion injury (IR injury) [68, 69]. A recent study identified a new pathway underlying IR injury through impairing FUNDC1-mediated mitophagy. CK2*α* is upregulated by IR injury which leads to the inactivation of FUNDC1 and inhibited protective mitophagy [68]. FUNDC1 downregulation also contributes to pressure-overload heart failure. *α*-LA treatment can activate FUNDC1-mediated mitophagy and attenuated cardiac injury [67].

Receptor-mediated mitophagy is also involved in the process of cell differentiation. A recent research revealed that BNIP3L/NIX and FUNDC1-mediated mitophagy, rather than PINK1-Parkin mediated mitophagy, is a crucial regulator of cardiac progenitor cell differentiation. In the process, receptor-mediated mitophagy facilitates proper mitochondrial network reorganization of the cells, and abrogating the pathway can cause abnormal mitochondrial morphology and impaired mitochondrial function [70].

## 4. Mitochondrial Dynamics

As early as 1914, M.R. Lewis and W.H. Lewis observed the continuous fusion and fission of mitochondria in the cultured chicken embryo tissues, which they called mitochondrial dynamics [71]. Since then, mitochondrial fusion and division have been observed in almost every species and cell type, except for adult cardiomyocytes. The special structure in mature cardiomyocytes causes their mitochondria to be relatively static, that is why the fusion and fission of cardiomyocytes and mitochondria cannot be directly observed [72]. However, compelling evidence has proved that mitochondrial dynamics not only exist in adult cardiomyocytes, but also play a crucial role in the MQC of the cells. Interfering with or blocking mitochondrial fusion and division can have a significant impact on cardiomyocytes [73].

The fusion and fission of mitochondria are mediated by multiple large dynamin-like GTPases. In mammals, the mitofusins (Mfn1 and Mfn2) and optic atrophy factor 1 (Opa1) mediate the fusion of the outer and inner mitochondrial membranes, respectively, while Drp1 mediates mitochondrial fission. The combined effect of these proteins determines the balance between fusion and fission of mitochondria. Cardiac-specific loss-of-function studies of the mitochondrial dynamic proteins mentioned above revealed the vital roles for mitochondrial fusion and fission in cardiomyocytes. Knockout of either of the Mfn1/2 or Drp1 induces embryonic lethality in mice [74, 75].

### 4.1. Mitochondrial Fusion

The fusion of mitochondria requires merging of both OMM and IMM. Mfn1/2 molecules on two adjacent mitochondria form both homo-oligomeric (Mfn1–Mfn1 or Mfn2–Mfn2) and hetero-oligomeric (Mfn1–Mfn2) connection [76]. After the tethering of mitofusins, GTP hydrolyzation enables mitochondrial fusion. Finally, Opa1 mediates the fusion of the IMM of the two mitochondria.

The exact mechanism of Mfn1/2-mediated tethering of adjacent mitochondria remains elusive. In the traditional theory, Mfn1/2 traverses the OMM twice and both its amino and carboxyl terminal are exposed to the cytoplasmic side. The HR2 domain of the C-terminal mediates the tethering process through forming a dimeric antiparallel coiled-coil structure with HR2 domains of Mfn1/2 molecules on adjacent mitochondria [77].

However, the traditional theory is challenged by recent progress. Firstly, biochemical evidence confirmed the C-terminal of Mfn1/2 reside within the intermembrane space (IMS). This result revised the former understanding of Mfn1/2, proving they are single-spanning OMM proteins with C-terminal embedded in the OMM and IMS and N-terminal out the OMM [78]. Secondly, two recent researches proposed a new model of Mfn1/2-mediated tethering of mitochondria. According to this model, GTP binding can induce conformational change and promote GTPase domain dimerization, which mediates the tethering process. This model is based on crystal structures of dimerized partial Mfn1 proteins [76, 79]. Although it remains controversial, conformational change of Mfn1/2 during the tethering process has gained more and more recognition [76, 79–81].

### 4.2. Mitochondrial Fission

The division of mitochondria is mainly induced by a large dynamin-related GTPase Drp1 (Dnm1 in yeast). In contrast to Mfn1/2 and Opa1, Drp1 exists in cytoplasm. Under certain conditions, Drp1 is recruited to the OMM. Research shows that mitochondrial fission protein Fis1, mitochondrial fission factor (Mff), and mitochondrial dynein MiD49/51 are involved in the recruitment of Drp1, among which Mff plays a major role [82, 83]. Once recruited, Drp1 undergoes oligomerization and assembles around the mitochondria to form a ring structure. Afterwards, GTP binds to Drp1 and drives the contraction of the Drp1 loop, thereby splitting the mitochondria into two segments [84].

## 5. The UPS Regulates Mitochondrial Dynamic Proteins

Expression levels of the mitochondrial dynamic-related proteins collectively determine the balance between mitochondrial fusion and fission. The regulatory mechanisms of these proteins exist at multiple levels [85]. As described above, the UPS regulates the degradation of OMM proteins, in which mitochondrial dynamic-related proteins are also included. Through the degradation of these proteins, the UPS serves as the major mechanism regulating OMM mitochondrial dynamic proteins at protein cleavage level, which in turn determines mitochondrial fusion and fission.

As a E3 ligase, Parkin regulates levels of mitochondrial dynamic proteins. At the same time, it also plays a crucial role in mitophagy, facilitating the close cooperation between mitochondrial dynamics and mitophagy, which will be described in detail in the next part. Several other E3 ligases were shown to have an apparent influence on mitochondrial morphology under various stimuli, including Glycoprotein 78 (Gp78), mahogunin ring finger-1 (MGRN1), HUWE1, and MARCH5 (also known as MITOL), among which MARCH5 is the most studied [86].

MARCH5 belongs to the RING finger-containing proteins which include a large family of ubiquitin ligases involved in the proteasomal degradation of proteins. Residing on the OMM, MARCH5 was reported to have an apparent influence on mitochondrial morphology [87].

Several studies observed that MARCH5 mutant lacking ubiquitin ligase activity leads to mitochondrial fragmentation, indicating enhanced mitochondrial fission. Consistently, overexpression of MARCH5 promoted the elongation of mitochondria caused by increased level of mitochondrial fusion in a manner that depends on Mfn2 activity [88]. These results indicate MARCH5 has a vital role in regulating mitochondrial morphology, which is achieved by UPS degradation of mitochondrial dynamic proteins. A dominant-negative expression of Drp1 mutant counteracted mitochondrial fragmentation caused by MARCH5 mutation. Moreover, immunoprecipitation studies proved that MARCH5 interacts with Mfn2 and ubiquitinated forms of Drp1, which is supported by MARCH5 overexpression can increase turnover of Drp1. Another study pointed out that Mfn1 is also a substrate of MARCH5 [89]. In MARCH5 knockout cells, Mfn1 level is significantly increased, accompanied with highly interconnected and elongated mitochondria. The GTPase deficient mutant form of Mfn1 can counteract the effect of MARCH5 deletion, confirming Mfn1 as a substrate of MARCH5 [89].

Subsequent research finds that ubiquitylation of Mfn1 mediated by MARCH5 is increased during the G2/M phase of cell cycle. Then, Mfn1 is degraded through the proteasome-dependent way, and its expression level is downregulated [90]. MARCH5-mediated regulation of Mfn1 is also studied under mitochondrial stress condition caused by Antimycin A, which can block complex 3 of the respiratory chain. In this condition, Mfn1 is upregulated rapidly, but its level is also actively controlled. MARCH5-dependent ubiquitination of Mfn1 is also enhanced significantly, which promoted Mfn1 degradation through the UPS. This may act as an important mechanism that prevents mitochondrial aggregation and cell death caused by Mfn1 accumulation [91].

Taken together, the UPS plays an important role in regulating mitochondrial dynamic-related proteins under different conditions.

## 6. The Role of UPS in Mitophagy

The UPS and mitophagy are the two major proteolytic mechanisms in cell with different division of responsibilities. On the protein level, the UPS degrades soluble proteins by proteasome, while on the organelle level, mitophagy selectively degrades the whole mitochondria by engulfing the organelle with an autophagosome and fuses with lysosome. They also have something in common; the initiation of both ubiquitin-dependent type of mitophagy and the UPS relies on the ubiquitination of the desired substrates. However, different from ubiquitin-dependent modification of single protein of UPS, mitophagy depends on the massive ubiquitination of OMM proteins. These two mechanisms are closely related. They work in concert for the effective control of mitochondrial homeostasis. And the PINK1/Parkin pathway is the tie connecting them.

In the PINK1/Parkin-dependent mechanism of mitophagy, broad ubiquitination of OMM proteins occurs shortly after the recruitment of Parkin [92], which serves as a signal for both the recruitment of mitophagy receptors and the P97-dependent extraction of OMM proteins followed by their proteasomal degradation. Therefore, in the process of mitophagy mediated by PINK1/Parkin, some OMM proteins are degraded before the completion of mitophagy through the UPS pathway. Chan's group reveals that degradation of OMM proteins through the UPS is essential for mitophagy, and inhibition of the 26S proteasome can completely abrogate Parkin-dependent mitophagy [59].

### 6.1. Mfn2 Degradation via the UPS Facilitates Mitophagy

It is widely acknowledged that Mfn2 is a target of Parkin in response to depolarization [92–94]. Research shows that Parkin induces the ubiquitination of Mfn2 and results in their degradation by the UPS in a P97-dependent way [93]. This is confirmed by quantitative analysis of ubiquitylome during Parkin-dependent mitophagy [92], which shows a dramatic increase in ubiquitination of Mfn2 and a ~50% decrease in Mfn2 protein expression after mitochondrial depolarization.

Degradation of Mfn2 is thought to have a further influence on mitochondrial dynamics which plays an important role in facilitating mitophagy. The most common explanation for this is the elimination of mitofusins prevents the refusion of the isolated mitochondria with other healthy ones [93]. What is more, Mfn2 is also reported to act as a mitochondria-ER tether. UPS degradation of Mfn2 can also facilitate mitophagy by breaking the mitochondria-ER contact sites [95]. Indeed, studies have shown that Mfn2 degradation through the UPS is a prerequisite for Parkin-dependent mitophagy [59, 96].

### 6.2. UPS Degradation of FUNDC1 Regulates Hypoxia-Induced Mitophagy

A recent report found that the UPS-dependent degradation of mitophagy receptor FUNDC1 plays an important role in regulating hypoxia-induced mitophagy. The E3 ligase MARCH5 mediates the ubiquitination of FUNDC1 at lysine 119 directly, targeting it for subsequent proteasomal degradation. Through degrading FUNDC1, MARCH5 can desensitize mitochondria to hypoxia-induced mitophagy, while knockout of MARCH5 leads to enhanced mitochondrial sensitivity towards mitophagy-inducing stresses, as FUNDC1 degradation is significantly inhibited [97].

### 6.3. Regulation of Parkin-Dependent Mitophagy by DUBs

Recently, as components of the UPS, several deubiquitinating enzymes (DUBs) are found to play an important role in regulating the magnitude of Parkin-mediated mitophagy, including USP15, USP30, USP33, USP35, and USP36 [98]. DUBs can suppress the effects of Parkin not only by regulating the extent of ubiquitination of the substrates of Parkin but also Parkin itself [99]. Regulating DUBs may serve as potential therapies in PD patients through enhancing MQC.

USP30 and USP33 are only DUBs proved to be located at the OMM. UPS30 can preferentially remove Lys6-linked ubiquitin conjugates of its substrates, which is supported by crystal structure analysis of USP30. Research shows that USP30 can regulate Lys6-polyubiquitinated TOM20 on the OMM [100]. It is proposed that USP30 may regulate mitophagy by keeping mitochondrial ubiquitination below the threshold that can trigger mitophagy [100].

UPS33 is a newly identified DUB localized on the OMM. Different from USP30 which regulates substrates of Parkin, USP33 can directly and selectively deubiquitinate Parkin at Lys435 and preferentially remove K6, K11, K48, and K63-linked ubiquitin conjugates from Parkin. USP33 silencing dramatically increased K63-linked Parkin ubiquitination under mitochondrial depolarization. Simultaneously, Parkin recruited to the mitochondrial is increased, which leads to the enhancement of mitophagy [99].

## 7. Relationship between Mitochondrial Dynamics and Mitophagy

### 7.1. Mitophagy Is Preceded by Mitochondrial Fission: Is It the Case in Cardiomyocytes?

**Figure 2:**
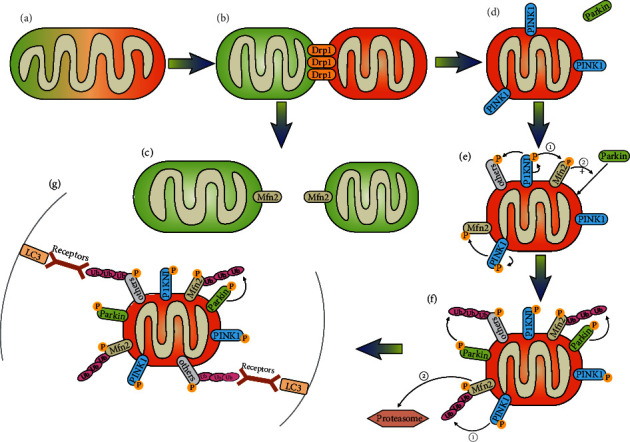
The role of mitochondrial fission and the UPS in PINK1/Parkin-mediated mitophagy. (a) Damaged mitochondria. (b) Though asymmetric fission of damaged mitochondria, two daughter mitochondria are produced. (c) The healthy daughter mitochondrial returns to normal mitochondrial population via fusion. (d) PINK1 accumulates on the outer membrane of the daughter mitochondrion with decreased membrane potential. (e) Accumulated PINK1 recruits and activates Parkin by phosphorylating its Ser65. (i) Other OMM proteins are phosphorylated as well. (ii) Phosphorylated Mfn2 promotes the recruitment of Parkin. (f) (i) Newly ubiquitinated proteins are phosphorylated by PINK1, which further promotes Parkin activity. (ii) Mfn2 is degraded by proteasome, preventing refusion of the isolated mitochondria with other healthy ones. (g) The mitophagy receptors bridge ubiquitinated OMM proteins with the forming autophagosome.

It is generally accepted that the mitochondrial fission is a necessary step previous of mitophagy [101–103]. On one hand, the size of mitochondria is too large to be engulfed by autophagosome at one time. Thus, mitochondrial fission favours mitophagy by dividing mitochondria into pieces of suitable size for engulfment. In contrast, mitochondrial fusion can protect the organelle from mitophagy [104]. On the other hand, mitochondrial fission is believed to separate the damaged part from the healthy part of mitochondria. Though asymmetric fission, two daughter mitochondria are produced. The one containing most part of healthy components of its parent organelle returns to normal mitochondrial population via fusion, while the other one with lower mitochondrial membrane potential (which is a mark of damage) is targeted for mitophagy through the PINK1/Parkin pathway (see Figure 2). This is supported by that Drp1 ablation leads to decreased mitophagy [103, 105].

However, in adult cardiomyocytes, whether mitochondrial fission is a prerequisite for mitophagy is questioned. The mitochondria of adult cardiomyocytes are organized in a highly structured and stable manner. Moreover, the morphology of cardiac mitochondria initially fragmented. This leads to the question that whether mitochondrial fission is necessary for mitophagy in the cardiomyocytes [106].

Cardiac-specific knockout of Drp1 can be a way to find the answer. Mitochondrial fission is interrupted in Drp1-null cardiomyocytes. If mitophagy is suppressed, it will support the hypothesis that mitochondrial fission is a prerequisite for mitophagy. Otherwise, if the level of mitophagy in cardiomyocytes is not reduced after Drp1 knockout, it indicates that mitochondrial fission is a necessary step previous of mitophagy.

Consistent with the latter one, Song et al. reported that cardiomyocyte-specific Drp1 knockout increased mitophagy, which resulted in a generalized loss of mitochondria [106]. This is supported by four evidences: (a) Mitochondria are observed inside cardiomyocyte autophagosomes in Drp1-knockout hearts. (b) Mitophagy markers (mitochondrial p62 and mitochondrial LC3-II) were increased significantly in Drp1-knockout hearts, indicating increased myocardial autophagosome-mitochondria interactions. (c) In cultured murine embryonic fibroblasts (MEFs), mcherry-Parkin was found clustered at mitochondria after Drp1 deletion, while in control groups, mcherry-Parkin was homogenously distributed throughout the cytosol. This indicates that Parkin-mediated mitophagy is increased. (d) After Drp1 deletion in MEFs, increased mitochondrial engulfment was observed [106]. Thus, the observation of Song's group supports that mitochondrial fission is not indispensable for mitophagy in cardiomyocytes. It seems the mitochondria are small enough for engulfment even without fission. And they proposed that increased mitophagy in Drp1-knockout cells is caused by mitochondrial permeability transition pore (MPTP) activation.

However, there are also contradictory results. The study of Ikeda and co-workers found mitophagy is inhibited following cardiac-specific Drp1 knockout [105]. They firstly observed that Drp1 mediates mitochondrial fission in response to glucose deprivation (GD). Then, by using mitochondria-targeted Keima fluorescence, they found Drp1 downregulation blocked the significant increase of mitophagy in cardiomyocytes under the condition of GD, suggesting that Drp1 is necessary for stimulating mitophagy. This is further proved by electron microscopic analysis, showing drp1 downregulation remarkably reduced the number of autophagosomes selectively containing mitochondria.

Taken together, whether mitochondrial fission serves as a premise for mitophagy in cardiomyocytes is still controversial. The reason behind this contradiction can be attributed to the difference of experiment models, conditions, and ways to measure the level of mitophagy.

### 7.2. Mfn2 Mediates Only One Process at a Time: Mitochondrial Fusion or Mitophagy

Mfn2 is the key molecular mediating mitochondrial fusion, simultaneously, and as a substrate of PINK1, it also plays a critical role in PINK1/Parkin-dependent mitophagy. Thus, the multifunctional protein Mfn2 acts as an important tie that links the two processes together.

According to a study of Chen and Dorn, in the process of PINK1-Parkin-mediated process, PINK1 can phosphorylase Mfn2 on Thr111 and Ser442, which then serves as a Parkin receptor [107–110]. This is supported by the fact that cardiomyocyte-specific and neuronal-specific knockout of Mfn2 can both result in deficiencies of Parkin translocation to depolarized mitochondria [111]. In addition, glutamic acid (E) substitution of Thr111 and Ser442 which mimics phosphorylation of Mfn2 is sufficient to induce Parkin translocation independent of PINK1. On the contrary, Thr111 and Ser442 phosphorylation can be prevented by alanine (A) mutations, and this abolished Mfn2-Parkin binding.

What is more, research of cultured cells expressing Mfn2 AA or Mfn2 EE provided another important conclusion that the Mfn2 function of binding Parkin and inducing mitochondrial fusion are mutually exclusive. Phosphorylated Mfn2 or Mfn2 EE can act as a receptor of Parkin but cannot mediate fusion. On the contrary, unphosphorylated Mfn2 or Mfn2 AA cannot recruit Parkin but serves as major mediators of mitochondrial fusion.

Thus, phosphorylation of Mfn2 by PINK1 can serve as a mechanism preventing refusion of the damaged mitochondria with healthy population. This mechanism functions even earlier than the above reviewed degradation of Mfn2 via the UPS system after Mfn2 is attached with ubiquitin by Parkin.

### 7.3. The Main Mechanism behind Cardiomyopathy of Mfn2-Null Heart

So far, most studies focusing on mitochondrial dynamics are based on the results of manipulation of mitochondrial dynamic-related genes. Mfn2 or Mfn1/2 combined deletion is one of the most frequently used methods. As introduced above, Mfn2 participates in both mitochondrial fusion and mitophagy. In other words, knockout of Mfn2 will have a profound influence on both processes simultaneously. This brings up a problem: which factor plays the major role in the pathological changes of cardiomyocytes induced by Mfn2 knockout, the impairment of mitochondrial dynamics or mitophagy? There are a lot of arguments and discussion surrounding this question.

For instance, Chen and Dorn reported that cardiac-specific Mfn2 knockout leads to heart failure. They attribute the consequence to impaired mitophagy, but not to suppressed mitochondrial fusion [107]. In contrast, in another study investigating the role of mitochondrial dynamics in the pathogenesis of diabetic cardiomyopathy, imbalanced mitochondrial dynamics is considered to be the major reason for cardiomyopathy after Mfn2 knockdown. This is based on the observations that fusion activator M1 restored mitochondria, while fission activator carbonyl cyanide 4-trifluoromethoxyphenylhydrazone (FCCP) blunted protective effects of Mfn2 upregulation in cultured cardiomyocytes treated by high glucose and high fat [12].

Intriguingly, in some cases, Mfn2 AA and Mfn2 EE reviewed above can be used as a tool to distinguish between the consequences of Mfn2-mediated mitochondrial fusion and mitophagy. As Mfn2 AA cannot be phosphorylated and function as a receptor for Parkin, PINK1/Parkin mitophagy is impaired. But it can still work normally to mediate mitochondrial fusion. By using Mfn2 AA, Gong et al. bypassed the disturbance to mitochondrial fusion that would be brought by Mfn2 knockout. They find that Mfn2 AA mice closely phenocopied perinatal cardiac myocyte of Parkin deletion, proving mitochondrial maturation in perinatal hearts relies on Parkin-mediated mitophagy [109]. Perhaps in the future we can compare the phenotype between Mfn2 ablation and Mfn2 AA mice, as the difference between them is caused by interrupted mitochondrial fusion, with the level of mitophagy being the same.

## 8. Conclusions

MQC is critical to the health of cardiomyocytes, and its dysregulation is closely related to the occurrence and development of cardiovascular diseases [112–115]. As three major components of MQC system, the UPS, mitochondrial dynamics, and mitophagy are interconnected and well-orchestrated, forming a triangle interplay.

The UPS serves as the major mechanism degrading soluble proteins in the cell. It also plays a critical role in degrading proteins embedded in the OMM and resides in the mitochondria. Thus, mitochondrial dynamic-related proteins and mitophagy-related proteins are also substrates of the UPS. Through degrading these proteins, the UPS can act as a critical regulator of mitochondrial fusion, fission, and mitophagy. During Parkin-mediated mitophagy, broad ubiquitination of OMM proteins can activate the UPS at the same time. Protein degradation via the UPS is vital for the subsequent progress of mitophagy. Mitochondrial dynamics and mitophagy are closely related processes. Mfn2 may serve as key molecular linking them together.

There are still several points we need to pay attention to in future research. Firstly, we need to realize the disadvantages of gene knockout studies, especially the ones expressing multifunctional proteins. For example, Mfn2 ablation can have a dramatic influence on both mitochondrial fusion and mitophagy and even to some other potential processes that have not been realized by now. Thus, it is inappropriate to simply attribute the phenotype of animal model to change mitochondrial dynamics. Other influenced processes brought by Mfn2 deletion must be considered at the same time. Secondly, more advanced experimental strategies and methods are still needed for understanding the role of MQC in cardiac cells, such as the methods to trace mitochondrial dynamics and network *in vivo*. Thirdly, we need to consider how to transfer the results of basic research into clinical practice, which is our ultimate goal.

In conclusion, our review summarizes the new processes of the three major MQC mechanisms and delves into the relationship between them. We provide a new perspective to understand MQC in cardiomyocytes, showing that as parts of MQC mechanisms, the UPS, mitochondrial dynamics, and mitophagy interact with each other and cooperate well, forming a triangle interplay. As MQC is vital to the health of cardiomyocytes, recovery and maintenance of its normal function may be a promising target for the treatment of CVD. There is a still long way to go to fully understand the mechanisms of MQC, which is the basis for its clinical application.
